# Exploring the views of Singapore junior doctors on medical curricula for the digital age: A case study

**DOI:** 10.1371/journal.pone.0281108

**Published:** 2023-03-02

**Authors:** Humairah Zainal, Xin Xiaohui, Julian Thumboo, Fong Kok Yong

**Affiliations:** 1 Health Services Research Unit, Singapore General Hospital, Singapore, Singapore; 2 Department of Rheumatology and Immunology, Singapore General Hospital, Singapore, Singapore; 3 Duke-NUS Medical School, National University of Singapore, Singapore, Singapore; University of Newcastle, AUSTRALIA

## Abstract

This study aims to explore the perspectives of medical trainees on the impartation of digital competencies in Singapore’s medical school curricula. It also considers how the medical school experience can be strengthened in order to bridge potential gaps in the integration of these competencies in the local curricula. Findings were drawn from individual interviews with 44 junior doctors from Singapore’s public healthcare institutions including hospitals and national specialty centers. House officers and residents from different medical and surgical specialties were recruited using purposive sampling. Data was interpreted using qualitative thematic analysis. The doctors were in their first to tenth year of post-graduate training. Thirty of them graduated from the three local medical schools whereas 14 others were trained overseas. Overall, they felt insufficiently prepared to utilize digital technologies in view of their limited exposure to such technologies in medical school. Six key reasons were identified: lack of flexibility and dynamism within the curriculum, dated learning style, limited access to electronic health records, gradual uptake of digital technologies in the healthcare sector, lack of an ecosystem that promotes innovation, and lack of guidance from qualified and available mentors. Equipping medical students with skills relevant to the digital age would benefit from a concerted effort from multiple stakeholders: medical schools, medical educators and innovators, as well as the government. This study bears important implications for countries that seek to bridge the ‘transformation gap’ brought about by the digital age, which is defined as the sharp divergence between innovations that healthcare providers recognize as important but for which they feel insufficiently prepared.

## Introduction

Healthcare is undergoing rapid digital transformations worldwide. This trend occurs against the backdrop of increasing technological advances in cloud computing, mobile, big data and social networks from the year 2000 onwards [1]. The subsequent rise of digital technologies since 2010, which is characterized by Artificial Intelligence (AI), Internet of Things (IoT), Machine Learning (ML) and the like, has to some extent, influenced clinical processes around care delivery and is expected to be a game-changer for the healthcare industry [1]. In view of these transformations, medical trainees need to be trained in relevant digital competencies needed in clinical practice.

Although some medical schools in developed countries have introduced a few aspects of these technologies in their curricula, studies have reported that there is a perceived inadequacy of digital competencies among medical students and junior doctors in these new developments. For example, in a self-administered survey of 451 medical students from 39 European countries such as Germany and Portugal on their perceived knowledge of digital health, more than half or 53.2% of them evaluated their eHealth skills as ‘poor’ or ‘very poor’, citing the lack of education to be the reason for this [2]. This perceived inadequacy, also known as the ‘transformation gap’, refers to the sharp divergence between innovations that health providers recognize as important but for which they feel insufficiently prepared [3].

While some existing studies focus on training gaps within the medical school curriculum [4–7], this paper contributes to the extant literature by interrogating issues that may lie in the curriculum as well as healthcare system using Singapore as a case study. Despite advances in digital technologies in both the economic and healthcare sectors, and high standards of medical education [8–10], there is a perceived disconnect between contemporary clinical practice and digital competencies among medical graduates in Singapore [11]. The latter include skills in data privacy and regulations, as well as data analysis and interpretation [11]. Although there have been initiatives to introduce courses related to digital technologies in the core curriculum, such as informatics and digital health, they vary in content and duration across the three medical schools in Singapore. Current training in the application of these skills to clinical practice is also limited [12].

Hence, this paper seeks to address two research questions: (i) What are the broader structural, cultural and institutional factors that may contribute to training gaps? and (ii) How can the medical school experience be strengthened to keep up with advances in technology? It does so by exploring the perspectives of physicians with one to 10 years of clinical experience. These physicians, who are medical graduates who have attained their basic medical degree (M.B.B.S) but have yet to embark on advanced specialty training, are also known as ‘junior doctors’ in Singapore [13]. Uncovering their views would enable an analysis of how the medical school curricula may have evolved over time, reveal potential gaps in the healthcare system and how medical trainees can be better supported when utilizing digital technologies in clinical care. Overall, it bears important implications for countries that seek to bridge the ‘transformation gap’ brought about by the digital age.

## Materials and methods

### Sample and setting

The consolidated criteria for reporting qualitative research (COREQ) were followed in the reporting of this study [14]. The completed checklist is found in S1 Table. Data was collected from September 2020 to July 2021 through semi-structured individual interviews with junior doctors. Purposive and convenience sampling was used to recruit house officers and residents from different surgical and medical specialties such as Orthopedic Surgery, Internal Medicine and Ophthalmology. To examine the extent to which the curricula have evolved over the years, junior doctors with different years of clinical experience were recruited; mainly, those with one to five years of experience and those with six to ten. Doctors who were trained in different countries were also recruited, as comparing the views of those trained locally and overseas was designed to help us identify the strengths and gaps of Singapore’s medical curricula in preparing students for the digital age. The doctors were recruited through email announcements, department heads and snowballing technique. Waiver of review was granted by SingHealth Centralised Institutional Review Board (Reference Number: 2020/2880).

### Data collection

Participants read a study information sheet and had their questions answered before email consent was documented and basic demographic data was collected. All the interviews were conducted in English by researcher HZ over Zoom. Each interview lasted approximately 30 minutes. The interview questions addressed digital technologies listed by Forbes as the latest technology trends that would transform medicine and healthcare in 2020, which included AI, ML and robotics [15], their medical school curriculum and their exposure to any new forms of technology in clinical practice (S2 Table). These were formulated based on the research questions established for this study, as stated in the Introduction, as well as consensus with the team.

During the interview, to avoid the pitfall of under-reporting where the participants’ ability to recall their medical school curricula accurately would limit their responses, we mentioned digital technologies that may be relevant to clinical practice based on our literature review of technological trends that will transform healthcare, as well as search of courses offered by the schools as listed on the schools’ websites. For example, for participants who graduated from National University of Singapore’s Yong Loo Lin School of Medicine (YLL), we asked about their exposure to informatics, if any. This question proved useful as it provided us with insights on the coverage of courses related to digital technologies in the curricula. Mentioning some digital technologies was also necessary because most participants were uncertain about what constituted ‘digital technologies.’ Hence the need to provide them with relevant examples.

In reviewing issues of reflexivity in qualitative research [16], we were made aware of potential researcher bias that might be present due to the established professional relationship between the Principal Investigator and research participants. The interviewer, having no prior relationship with any of the participants, was an important measure to counter the threat of bias. To prevent this from influencing our research, we assigned code identifiers to each participant to ensure anonymity. Codes that begin with ‘HO’ refer to house officers, ‘JR’ refer to junior residents while ‘SR’ stand for senior residents. These acronyms are followed with the specialty that the participants were posted to at the time of interview, and the country where they took their medical degree.

### Data analysis

The interviews were transcribed verbatim by a transcriber. The transcripts were reviewed by HZ and Principal Investigator FKY to ensure transcription accuracy. They were analyzed using NVivo 12 concurrent with conducting other interviews until data saturation was reached. HZ and FKY read the transcripts independently and adopted an inductive thematic analysis approach when evaluating the data to draw common and shared meanings among participants [17]. Thematic analysis proved relevant because it involves ‘more than description and categorization, but not extending so far as to develop theory’ [17]. Coding frameworks and themes were developed iteratively using Braun and Clarke’s (2006) six-step process: familiarizing with the data, generating initial codes, searching for key themes, reviewing themes, defining and naming themes and producing the report [18]. HZ and FKY coded the data independently before resolving discrepancies in assessment through consensus.

Data from junior doctors trained in different specialties and who graduated from medical schools were collected to ensure accuracy of results. Credibility was maintained by cross-checking the information provided by the respondents with other key stakeholders. The latter included deans and vice deans of education of local medical schools, clinical educators and other senior consultants working in Singapore’s public healthcare institutions including hospitals and national specialty centers.

## Results

Forty-four junior doctors (23 females) from 18 different specialties and 11 public healthcare institutions participated in the study. The doctors, aged between 25 and 35 years, were in their first to tenth year of post-graduate training. Thirty of them graduated from the three local medical schools whereas 14 others were trained in the United Kingdom, Australia and Malaysia (Table 1).

**Table 1:** Demographic of participants.

Profile	Number of participants
Gender	
• Male	21
• Female	23
Age range	
• 21–30 years	30
• 31–40 years	14
Years of clinical experience (post-graduation)	
• 1–5 years	31
• 6–10 years	13
Countries where participants attended medical school	
• Singapore	30
• United Kingdom	7
• Australia	6
• Malaysia	1
Specialties	
• Medical	30
• Surgical	14

The findings demonstrate that the skills deemed lacking in the respondents’ curricula were neither differentiated by their years of clinical experience nor the schools they graduated from. As shown in Table 2 below, the curriculum has not evolved significantly in the last 10 years, with respondents who graduated within the past 5 years receiving only a brief exposure to digital technologies such as informatics, VR and telemedicine in their core curriculum while those who graduated earlier had none. Any attempts to initiate medical innovation projects often took place outside of the formal school curriculum, which we define as the structured education that takes place within the classroom setting. Moreover, despite the acceleration in the uptake of digital technologies such as AI, analytics and robotics in Singapore’s healthcare system during the COVID-19 pandemic [11], the respondents reported that their exposure to these developments was almost non-existent. Where technologies were used, they were usually meant for communication with other healthcare providers and not for clinical practice.

**Table 2:** Summary of respondents’ reported exposure to digital technologies in the medical school curriculum.

Years of Clinical Experience (Post-graduation)	Number of Participants	Amount of Exposure to Digital Technologies in Formal Curriculum	Amount of Exposure to Digital Technologies in Informal Curriculum
1–5 years (Graduated between 2016 and 2021)	31	NUS’s YLL students (Class of 2020) (n = 6): • Exposed to a core module in third year where students learnt basic informatics and statistics • Brief, 10-minute exposure to Virtual Reality in core curriculum • Brief exposure to 3D technology in anatomy in the last few clinical postings Students from Nanyang Technological University’s Lee Kong Chian School of Medicine (LKCMedicine) and Duke-NUS Medical School who graduated within the last 5 years (n = 10): • Brief exposure to telemedicine lasting about two hours during GP posting* Graduates from Australia who graduated within the last 5 years (n = 3): • Encountered telemedicine during rural GP posting, where the use of telemedicine was prevalent	NUS’s YLL student (Class of 2020) (n = 1): • Participated in one-year NUS Medical Grand Challenge, a student-led medical innovation program introduced by YLL in 2017 that teams up medical students with students from two other faculties to drive innovations in healthcare and solve unmet healthcare needs within a year [19].
6–10 years (Graduated between 2011 and 2015)	13	None had any exposure to digital technologies	None

*Singapore students’ exposure to primary care settings is limited to begin with -for details on the length of posting in GP clinics, refer to Zainal and Smith 2020 [20].

The above indicates that there may be persisting gaps that have yet to be addressed by many medical schools around the world- a point that will be elaborated below. The sentiment that the medical school curricula were largely didactic and memorization-based was also shared by the respondents regardless of the country where they attended medical school. For some who graduated from the UK, their schools started incorporating digitalization-related topics in the core curriculum, such as data analytics and coding, only after they graduated. Other courses such as computer science in the context of medicine were offered only towards the end of their medical school. Even so, they were taught as electives and not part of the core curriculum. Gender was also not a factor in influencing the respondents’ amount of exposure to digital technologies. An elaboration of the respondents’ experiences with these technologies is presented below.

In view of the limited exposure to digitalization in their medical school curriculum, our respondents generally felt insufficiently prepared to utilize digital technologies in medicine. They attributed this to six key reasons, which included:

### Lack of flexibility and dynamism within the curriculum

There was the sentiment that there is a disconnect between contemporary practice and the medical school curriculum, which some respondents such as SR-07 and HO-10, quoted below, perceived as lacking in flexibility and dynamism.

In medical school, we were given a set of rules to follow but in real world medicine, that’s never the case. Sometimes, patients need flexible, individualized treatment. I think the problem lies in medical school being too didactic. Students are not taught how to be flexible and innovative. [SR-07, General surgery, Australia]

In medical school, a lot of the learning is theoretical. Everything is very safe, very structured, and you seem to target patients’ symptoms and medical issues one-by-one. But when you are in the wards, it is different; your patient comes in with multiple problems. Things are more dynamic. [HO-10, General medicine, Australia]

The excerpts prove the need to disrupt traditional educational structures and to strengthen the link between education and practice. The sharing by HO-10 also reinforces the importance of the medical school curriculum to remain relevant with contemporary healthcare needs. This is especially crucial given the growing healthcare needs of Singapore’s ageing population with multiple morbidities.

### Dated learning style

Other respondents reiterated the lag between the existing curriculum and evolving trends in clinical settings by highlighting the huge emphasis placed on rote memorization of medical information. On the other hand, digital competencies such as interpreting data on health-related smartphone applications and searching for relevant medical information on digital platforms were not emphasized as much, as cited below:

Medical school education was a bit archaic where we just memorized everything. In reality, a lot of patient management is quite automated, like choosing appropriate treatment, what to order and how to monitor patients. So, there are already structured protocols to consider… Healthcare apps could be useful in tracking the progress of some patients but then, how do you interpret the data? [JR-11, Rheumatology, Singapore]

Rote memorization of a whole list of things is less useful nowadays. It is useful to know the broad principles but realistically, if you do not know, you can still go back to searching it. So what ought to be emphasized more is knowing how to look for information, for example, being able to digest relevant papers and know the implications for clinical practice. [JR-09, Neurology, UK]

Skills in interpreting data from health apps constitute part of broader competencies needed for digital health, a field of medicine that broadly constitutes technologies such as electronic health records (EHRs), telemedicine, wearable devices, health information technology (IT) and personalized medicine [21]. Indeed, studies have shown that medical students, medical school faculty and leaders perceive practical training in data management including big data analysis, data sharing and data security as useful for students [2, 4, 22]. Being equipped with knowledge of the ethical and legal aspects of using digital health technologies as well as communication skills in advising patients to use digital health tools are also considered important [4]. However, just like the medical faculties in countries such as Germany [4], courses on digital health vary in scope and content across Singapore’s core medical school curricula.

Furthermore, skills in searching for relevant medical information has been recognized as important in the need to obtain trustworthy and validated information for patients as well as to reduce burnout among doctors that stem from information overload [23]. To equip students with these skills, training in new content areas such as metacognition, data science, informatics and AI are needed [23, 24]. The need for medical schools to reduce emphasis on memorization of medical information in order to devote more time for these skills has been acknowledged by scholars such as Cutrer et al (2021), Paranjape et al (2019) and Park et al (2019). However, while schools in other developed countries such as the USA have begun to adjust aspects of educational programming in the acknowledgment of rapid expansion of information [24], there remains a lack of scaffolding by medical schools in Singapore to explicitly teach students how to leverage information resources for clinical care.

### Limited access to EHRs

Although students were introduced to EHRs used in the hospitals, their lack of access to those used in actual clinical settings interfered with their acquisition of patient charting and medication skills as well as smooth transition to housemanship:

During the Student Internship Programme (SIP), we learnt about the computer systems but our accounts were different from the house officers’. So, there were a lot of things we could not do, like ordering medication. Our notes were also slightly different. There were some we could not access, so we could not write referral letters and all that. When I first started housemanship, I was struggling with all the different software- they are all very different. Took me weeks to get familiarized. [HO-02, Orthopaedics, Singapore]

It would be helpful if we were exposed to the records early enough and given the same account as the house officers’ during the SIP. It would also be good to have a more streamlined process or portal across all hospitals where we use the same software. [HO-05, Paediatrics, Singapore]

The excerpts reflect the importance of providing an authentic learning environment for students in order to enhance practice satisfaction and better prepare them for future clinical practice. Indeed, studies have shown that when students experience firsthand the utility and challenges of using health IT, they are able to hone their clinical decision-making skills, initiate quality improvement and interpret lab results found in patients’ records, among others [25]. Additionally, creating a single type of EHR across multiple training sites would also help to reduce training burden for students.

### Gradual uptake of digital technologies in the healthcare sector

Some interviewees also attributed their sense of inadequacy in utilizing digital technologies to the perceived gradual uptake of emerging technologies in healthcare:

We have only gone digital in terms of x-rays, results and documentation. Even so, it took us 20 over years. It is a very slow process. A lot of the things we are doing are actually still very old. [JR-10, Internal Medicine, Singapore]

Others pointed out that the acceleration of digital technology uptake in Singapore’s healthcare system would only be possible with a strong IT infrastructure, which seems to be lacking in the current system:

My main gripe is IT support. When it’s downtime, it’s really disruptive and nobody knows how to remedy. There was once when my account was just hanging for a good six hours. That also means the patients are just lying in recovery without any plans and nobody knows what to do with them. So if you want to be advanced, the IT system has to be reliable and advanced as well. [SR-03, Orthopaedics, Singapore]

The excerpt by JR-10 reiterates our observation that despite Singapore’s digital competitiveness, the healthcare sector is still lagging behind in terms of technological uptake, especially in the realm of clinical care. While reasons for the lag in digital transformation of healthcare may include complexities of the healthcare system and the high stakes involved when technology is adopted in critical decision-making [26], a lack of active adoption of basic technology is also a major barrier to the uptake of more advanced forms of technology.

Additionally, SR-03’s sharing highlights the importance of strengthening the IT infrastructure, as gaps in the infrastructure will interfere with patient care. Studies have also reported that inefficiencies in the healthcare’s IT system have caused burnout and job unsatisfaction among junior doctors [27]. On the other hand, a fully integrated IT system would improve access to information for patients, carers and healthcare professionals [28]. Hence, limitations in IT infrastructure prove to be another barrier that need to be addressed effectively by relevant stakeholders.

### Lack of an ecosystem that promotes the culture of innovation

Some respondents opined that there is a lack of ecosystem within local healthcare institutions that promotes a culture of innovation among medical trainees. They attributed this to the lack of time brought about by a heavy workload and of an open mindset towards medical innovation in general, as exemplified by JR-10 and SR-01, respectively:

Our healthcare system overvalues efficiency and ability to take a heavy workload, so, most of the time, junior doctors are just stuck doing work. The system needs to be less of a pressure cooker so that doctors will have time to explore other interests (beyond clinical work). [JR-10, Internal Medicine, Singapore]

When I was trying to put up a grant for a medical innovation project, I met with some resistance from those in the senior level who questioned why I was doing that project, who was going to access the online portal I wanted to create. They believed the patients and doctors are not going to use it, but I think change has to start from somewhere. If we are willing to start making a change, that is how things will advance [SR-01, Obstetrics and Gynaecology, Ireland].

The above excerpts demonstrate that while there may be interest among medical trainees to engage in medical innovation projects, several systemic barriers remain. There has to be a mindset shift in terms of how the healthcare system treats its manpower resources, as it currently appears to tie the worth of labour to productivity, which may inadvertently reduce the creativity and diversity of talents within the healthcare workforce. The attitudes of healthcare leaders towards projects involving technology or innovation should also change given their role in developing and maintaining the governance and standards of doctors. Moreover, broader digital technology initiatives by medical schools and healthcare institutions should trickle down to the core undergraduate curricula, as current sustained efforts to train budding physicians in digital technologies are largely limited to postgraduate programs [29].

### Lack of guidance from qualified and available mentors

Those who were interested in the specifics of digital technologies pointed to the lack of perceived expertise and guidance from seniors who were involved in technological innovation projects:

When I asked a Consultant for details on how a triage that involved AI worked, all she could tell me was, there was some AI involved. This is what happens when the average doctor who may not be that interested in these technologies, work on such projects. [JR-10, Internal Medicine, Singapore]

Some respondents also highlighted that their limited training in digital technologies used in the wards, coupled with a lack of proper guidance, proved challenging in their clinical practice, as illustrated by SR-01 below:

We were not trained to perform bedside ultrasounds so when I first started out in O&G, whenever a patient came to the emergency clinic, if it was acute abdomen, we would send them to the radiology department or reschedule for them to come back to outpatient clinic where they can get a formal scan… and when I was a medical student in Ireland, we weren’t really allowed to perform any procedures, like setting IV cannulas for patients. So, when I first started out as a HO, I absolutely had no idea how to set a plug. I had a nurse to teach me that. It’s often difficult to find a senior to supervise you because they will be really busy. [SR-01, Obstetrics and Gynaecology, Ireland].

The above excerpts reiterate the need for a mentoring system and formal training in digital technologies for trainees. In addition to equipping trainees with the necessary competencies, clinical educators and mentors themselves have to be knowledgeable in these fields. The availability of qualified mentors would also help to foster a culture of innovation among trainees.

The findings show that the gaps in the training of digital technologies within the medical school curricula are largely intertwined with those in actual clinical settings and should not be addressed in silos. Specifically, they demonstrate how medical education, the clinical setting and the healthcare system work as a continuous feedback loop that inform and enhance one another. In so doing, the study reiterates the importance of multiple stakeholders in strengthening the medical school experience.

## Discussion

The findings highlight that the junior doctors felt insufficiently prepared to utilize digital technologies in view of their limited exposure to such technologies in medical school. Six key reasons were identified: lack of flexibility and dynamism within the curriculum, dated learning style, limited access to EHRs, gradual uptake of digital technologies in the healthcare sector, lack of an ecosystem that promotes innovation, and lack of guidance from qualified mentors. By interviewing respondents from different medical schools, we found that perceived gaps in equipping medical students with relevant digital competencies were not unique to any particular school. Additionally, by obtaining feedback from junior doctors trained in diverse clinical specialties and medical schools, this paper has uncovered the perspectives of an important stakeholder in the healthcare system.

Furthermore, by exploring broader structural and cultural factors beyond the medical school that may contribute to the aforementioned gaps, this paper fills the dearth in literature on digital competencies in Asian medical schools. Past studies conducted in countries such as Hong Kong, Japan and Pakistan have mostly addressed curricula gaps through pedagogical approaches or improvements within medical schools [30–32]. Unlike these studies, our research contextualises the medical school curricula within the wider context of digital transformations of healthcare.

It appears that the six limitations and barriers to bridging the transformation gap faced by Singapore are also encountered by other developed countries. For example, institutional, logistical and structural barriers to the use of EHRs in clinical settings have also been experienced by some medical students in the USA [33]. The divergence between competencies taught in medical schools and those relevant to clinical practice has also been discussed in studies done in USA and Italy [7, 34]. Areas where digital skills and expertise are needed but that are lacking among the teaching fraternity are also common in the USA and Switzerland [35, 36]. Furthermore, the perception that healthcare lags behind other industries in the implementation of digital technologies has also been recognized by other studies [37, 38].

Yet, while some medical schools in countries such as Germany and Switzerland have made training in telemedicine, digital medicine and data science mandatory for its students [22, 36], these courses are largely taught as electives or only briefly within Singapore’s formal medical school curricula. Additionally, while a memorization-based and risk-averse curricula form a common challenge of most medical schools in developed economies [12, 39, 40], what forms a significant barrier to innovation and curricular implementation of digital topics in Singapore is its hectic clinical environment, which offers very limited time for its trainees to explore digital technologies and embark on innovation projects of interest. A study reported that the professional burnout rate among junior doctors in Singapore was higher than that in the USA despite both countries sharing similar residency programs and Accreditation Council for Graduate Medical Education (ACGME) guidelines [41]. Indeed, the study showed that long working hours and high job stress contributed to the residents’ high burnout rate and did not rule out the possibility of this being accumulated from their medical school training [41].

## Recommendations

Findings from the interviews have reiterated the importance of bridging the transformation gap in Singapore and of systematically collecting feedback from practicing doctors. This section offers recommendations to address each of the barriers.

### Encourage a shift in assessment of competencies and adaptive learning

In response to concerns about the lack of flexibility in the curriculum, medical schools need to reexamine the way in which students’ clinical competence is assessed. Currently, students are largely tested on their theoretical knowledge, and medical examinations are heavily focused on the basic sciences. A redesign of the curriculum could involve curriculum expansion and a review of assessment strategies. For example, there should be an increase in the assessment of creativity, critical thinking skills and concepts that would contribute to students’ understanding of digital technologies. Students should also be tested on the fundamentals of data science and topics related to digitalization in their final examinations.

Additionally, to address the concern about the lack of dynamism in the curriculum, medical schools need to equip students with adaptive skills. Cutrer et al (2021) have highlighted the value of a curriculum that gears learners to learn and innovate in response to practice challenges rather than one that focuses primarily on the delivery of content [24]. For example, at Harvard Medical School, which has reorganized its entire curriculum using a case-based collaborative learning model as opposed to a performance-oriented culture, students are valued for their reasoning skills rather than just for correct answers [24]. At Vanderbilt University School of Medicine, students are required to identify knowledge gaps in the course of patient care and present their findings to a clinical team that will assess their skills in information appraisal and application [24].

### Provide dedicated training in digital competencies

To ensure that the school curricula remain up-to-date and are aligned with the needs of the healthcare system, a dedicated training in digital competencies for clinical practice is needed. One way is to reduce the duration on basic sciences education, especially those that require students to memorize a barrage of medical information, and increase the time spent on educating them about the fundamentals of digital technologies instead. Research have shown that longitudinal curricula that span the duration of medical school would expose students to a wider range of skills [7, 4]. For instance, Hersh et. al. (2017) highlight that an undergraduate medical curriculum that includes a comprehensive set of competencies in clinical informatics would expose students not only to EHRs but also to telemedicine and personalized medicine as well as other useful skills in clinical decision support and quality improvement [7]. Likewise, Foadi et. al. (2021) show how integrating digital competencies longitudinally into the curriculum can equip students with skills such as handling of medical data, knowledge of digital infrastructure of the health system, application of data in patient care and preventive medicine, ethical and medico-legal basics and transformation processes in medicine brought about by digitalization [4].

To maximize effective implementation of the aforementioned skills, educational systems, program structures and objectives need to be revised in order to create new learning outcomes [7]. A systematic curricular reform would also require medical schools and healthcare professionals to incorporate digital competencies in their ongoing dialogue on medical education [42]. During clinical rotations, medical experts should consciously introduce students to relevant applications of digital technologies in practice.

### Expand students’ access to EHRs

To increase students’ access to EHRs, a concerted effort of multiple stakeholders is needed. At the regulatory level, medical innovators should create EHRs that are the same as those used in actual clinical settings while recognizing the need for protection of confidentiality and regulation of access for specific purposes. For instance, deidentified patient data can be used to encourage students to practice writing orders, entering notes, reviewing data and formulating a care plan, as has been adopted in the US [33]. At the institutional level, schools should devise policies on the placement of students’ notes that, while compliant with all regulations, balance the need for patient privacy, liability concerns and the need for students to become competent with EHRs [33]. At the national level, a move towards interoperability of systems that allow users to share data would facilitate students’ adaptation to new systems in different healthcare settings [33].

### Optimize digital technologies in healthcare

To address the perceived lag in the uptake of digital technologies in healthcare, hospitals and policymakers would first need to ensure that technological equipment and processes in clinical settings are up-to-date and operating at maximum efficiency. Hospitals would also benefit from obtaining medical innovators’ advice on ways to optimize digital technologies in diagnosis, treatment and overall patient care as well as reduce inefficiencies in poorly designed systems. A technology assessment committee could also be formed to develop clear policy guidelines that would enable medical students and healthcare professionals to utilize digital technologies safely and effectively without compromising patient care.

### Foster a culture of innovation

In response to the feedback on the lack of an ecosystem that promotes the culture of innovation among medical trainees, we recommend a better balance between the provision of high-quality care to patients and a more efficient management of manpower resources in healthcare institutions to allow time for doctors to pursue projects that involve digital technologies. Protected time to mentor trainees in medical innovation projects should also be offered to qualified candidates. For students interested in medical innovation projects, healthcare institutions could offer them dedicated training during a gap year in their clinical years. Additionally, relevant recognition in the form of awards or opportunities for collaboration with clinicians involved in digital technology projects should be given to trainees who have demonstrated sufficient potential.

### Provide mentorship to students

Lastly, to address trainees’ concern about the lack of guidance from qualified seniors, strong mentorship from competent medical educators is necessary. Interdisciplinary collaboration and networking among educators could help address the need for combined expertise in digitalization, medical education and medical science [36]. This would help to ensure the inclusion of competence from the widest range of specializations [36]. For doctors with little or no experience with digital technologies, they could undergo training via Continual Medical Education (CME) programs and learn from those outside of the medical community. All in all, proper mentorship from qualified educators is important as studies have shown that technological savviness among younger physicians does not necessarily translate into skills transferability to work with patients and in the professional medical setting [37, 43].

## Strengths and limitations

This qualitative study contributes to the growing body of international literature while informing us specifically about the skills pertinent to doctors in Singapore. The sample achieved diversity in specialties, healthcare institutions, years of clinical experience, schools attended, genders and ethnicities, which contributed to a rich data. By exploring how doctors trained in different medical schools from various parts of the world perceived the relevance of their medical school curricula for the digital age, this study identifies gaps in the local curricula and proposes recommendations in areas where improvements in undergraduate medical education can be made. Moreover, compared to past research that mostly focus on institutional inertia [2, 42, 44], our study reveals barriers beyond the medical school that have also contributed to these gaps.

As a qualitative study, generalizability may be compromised and as a cross-sectional study, tracking changes over time may be limited. Nonetheless, interviewing respondents with up to 10 years of clinical experience demonstrates that few changes have been made to the school curricula to prepare students for the digital healthcare landscape. Furthermore, stating in the recruitment email that the interview will be about digitalization in healthcare may have led to selection bias as those doctors who responded to the call for interview might be the ones who were interested in digital technologies or who have had experience working on projects related to such technologies. However, this did not skew the results because their responses were nuanced. Additionally, as Table 2 above has shown, the respondents’ experiences with digital technologies were limited. Their knowledge of these technologies was also superficial.

A potential line of future research would be to explore the views of other stakeholders such as policymakers, medical educators and medical technopreneurs on other barriers to substantive curricular reform. Furthermore, it would be worthwhile to gather the views of doctors working in private healthcare institutions, to highlight any differences between their exposure to digital technologies and those from the public sector. The qualitative feedback obtained from this study could also be used to develop a checklist or questionnaire that identifies gaps for specific digital competencies.

## Conclusions

Our study explored the reasons for the lag in Singapore’s medical school curricula in preparing students for clinical practice in the digital age from the perspective of junior doctors. It has shown that the main bottleneck in the implementation of a rigorous digital-centric curricula for a developed country like Singapore is not so much infrastructural but structural, cultural and institutional. While previous studies have mostly identified gaps within medical schools, our findings highlight that these gaps are also intertwined with current limitations within the clinical setting and healthcare sector. Hence, input from those outside of the medical school community, as well as continuing medical education among practicing physicians would be integral to honing the skills relevant to current and future practice. More broadly, this study highlights the need for healthcare institutions to leverage on Singapore’s competitiveness in digital transformation so that the digital skills imparted to medical trainees will be congruent with the needs of changing times. Ultimately, the clinical competencies of trainees can only be strengthened with stronger collaboration among various stakeholders.

## Supporting information

S1 Table Completed checklist of the consolidated criteria for reporting qualitative research (COREQ). (DOCX)

S2 Table Interview guide. (DOCX)
