# Supplementary material for: Exploring the views of Singapore junior doctors on medical curricula for the digital age: A case study
Source: PLoS One. 2023 Mar 2;18(3):e0281108. doi: 10.1371/journal.pone.0281108 (PMC9980755; doi:10.1371/journal.pone.0281108)
Supplement: S1 Table — (DOCX) [file pone.0281108.s001.docx]

Supporting information File 1

COREQ (COnsolidated criteria for REporting Qualitative research) Checklist

| **Topic** | **Item No.** | **Guide Questions/Description** |
| --- | --- | --- |
| Interviewer/facilitator | 1 | Humairah Zainal (HZ) |
| Credentials | 2 | HZ [Ph.D., Sociology], Xin Xiaohui (XXH) [M.Soc.Sci, Sociology], Julian Thumboo (JT) [MBBS, MMed (Internal Medicine), FAMS (Rheumatology), FRCP] and Fong Kok Yong (FKY) [MBBS, MMed (Internal Medicine), FAMS (Internal Medicine, Rheumatology), FRCP] |
| Occupation | 3 | HZ- Research Fellow, XXH- Senior Research Manager, JT- Senior Consultant, Rheumatology and Immunology, FKY- Senior Consultant, Rheumatology and Immunology |
| Gender | 4 | HZ- female, XXH- female, JT- male, FKY- male |
| Experience and training | 5 | HZ- Trained in qualitative research and methodology, educational background in Sociology. XXH- educational background in Sociology. JT- educational background in Medicine. FKY- educational background in Medicine |
| Relationship established | 6 | No |
| Participant knowledge of  the interviewer | 7 | Participants were informed about the study by Principal Investigator FKY through email and that HZ was the research fellow conducting this research before being contacted by HZ with details about the study. |
|  |  |  |
|  |  |  |
| Interviewer characteristics | 8 | No other characteristics were reported by the interviewer. |
|  |  |  |
|  |  |  |
| Methodological orientation and Theory | 9 | Coding frameworks and themes were developed iteratively using Braun and Clarke’s (2006) six-step process. |
|  |  |  |
|  |  |  |
| Sampling | 10 | Purposive sampling and snowballing. |
|  |  |  |
|  |  |  |
| Method of approach | 11 | Via email and department heads. |
|  |  |  |
|  |  |  |
| Sample size | 12 | 44 participants. |
| Non-participation | 13 | Not applicable. |
| Setting of data collection | 14 | Over Zoom (due to the physical and social restrictions brought about by the COVID-19 pandemic) |
| Presence of non-  participants | 15 | None. |
|  |  |  |
|  |  |  |
| Description of sample | 16 | Female= 52.3 %, male= 47.7 %, 21-30 years old: 68.2%, 31-40 years old: 31.8%, medical specialty= 68.2%, surgical specialty= 31.8% |
|  |  |  |
|  |  |  |
| Interview guide | 17 | Provided as supplemental material |
|  |  |  |
| Repeat interviews | 18 | None |
| Audio/visual recording | 19 | Zoom and audio recording |
| Field notes | 20 | Yes, made after interview to jot down fieldwork impressions |
| Duration | 21 | 30-60 minutes |
| Data saturation | 22 | Yes |
| Transcripts returned | 23 | No |

| **Topic** | **Item No.** | **Guide Questions/Description** |
| --- | --- | --- |
|  |  |  |
| Number of data coders | 24 | Two |
| Description of the coding  tree | 25 | Yes. On page 7 of the manuscript under ‘Data Analysis’. |
|  |  |  |
| Derivation of themes | 26 | Inductive, derived from the data |
| Software | 27 | NVivo 12 |
| Participant checking | 28 | No |
| Quotations presented | 29 | Yes |
|  |  |  |
|  |  |  |
| Data and findings consistent | 30 | Yes |
| Clarity of major themes | 31 | Yes. These include perceptions towards preparedness for clinical practice in the digital age, challenges in clinical practice that were caused by or that could be overcome by technology, and digital competencies that medical doctors need to have in this digital age. |
| Clarity of minor themes | 32 | Yes. These include suggestions on what medical schools, professional bodies and the government can do to help doctors optimize the use of digital technologies for future clinical practice. |

Source: Tong A, Sainsbury P, Craig J. Consolidated criteria for reporting qualitative research (COREQ): a 32-item checklist for interviews and focus groups. *International Journal for Quality in Health Care*. 2007. Volume 19, Number 6: pp. 349 – 357
