# Supplementary material for: Exploring the views of Singapore junior doctors on medical curricula for the digital age: A case study
Source: PLoS One. 2023 Mar 2;18(3):e0281108. doi: 10.1371/journal.pone.0281108 (PMC9980755; doi:10.1371/journal.pone.0281108)
Supplement: S2 Table — (DOCX) [file pone.0281108.s002.docx]

Supporting information File 2

Interview Questions

1. What are the clinical skills that a medical doctor should have?

2. Are there any clinical skills that have become less essential or relevant in this digital age?

3. Are there any clinical skills that have been fulfilled or transformed by digital technology?

4. Do you think the clinical skills taught in your medical school had adequately prepared you for clinical practice, especially in the modern setting?

5. Have you used or come across any new forms of technology yet in your clinical practice, such as robotics surgery, machine learning or AI?

6. Have you encountered any recurring challenges in clinical practice that could be overcome by digital technology? Otherwise, what type of digital technology would help to enhance your current field of practice?

7. Do you notice any changes or improvements in the use of technological devices used in general clinical practice in the last few years? If so, what are some of these changes or improvements?

8. In addition to the skills you have mentioned, what new clinical skills should a medical doctor have today and in the future especially in light of the digital age?

9. What aspect of digital medicine would you be interested in learning if given the opportunity to do so, and why?

10. What can these stakeholders do to help doctors optimize the use of digital technologies in the future: (i) medical schools, (ii) professional bodies such as Singapore Medical Association and Academy of Medicine, and (iii) the government.
